# Ionizable Lipid Containing Nanocarriers for Antimicrobial Agent Delivery

**DOI:** 10.1002/smsc.202400145

**Published:** 2024-08-13

**Authors:** Haitao Yu, Sampa Sarkar, Z. L. Shaw, Brendan Dyett, Xudong Cai, Sue Lyn Yap, Charlotte E. Conn, Aaron Elbourne, Calum J. Drummond, Jiali Zhai

**Affiliations:** ^1^ School of Science STEM College RMIT University Melbourne Victoria 3000 Australia; ^2^ School of Engineering STEM College RMIT University Melbourne Victoria 3000 Australia

**Keywords:** antimicrobial resistance, ionizable lipids, lipid nanoparticles, lyotropic liquid crystals, piperacillin

## Abstract

Antimicrobial resistance (AMR) poses a global health crisis demanding innovative solutions. Traditional antibiotics, though pivotal over the past century in combating bacterial infections, face diminished efficacy against evolving bacterial defense mechanisms, especially in Gram‐negative strains. This study explores self‐assembled ionizable lipid nanoparticles (LNPs) with the incorporation of two ionizable lipid components (one cationic, one anionic) in nanocarriers for advanced antimicrobial drug delivery of the broad‐spectrum antibiotic Piperacillin (Pip). Incorporating cationic ionizable lipid ALC‐0315, recognized as a functional lipid in the Pfizer‐BioNTech mRNA‐based SARS‐CoV‐2 vaccine, into LNPs allowed mesophase transition, pH responsiveness, and ionization behavior in acidic environments found in sites of bacterial infections, to be studied using synchrotron small angle X‐ray scattering, dynamic light scattering, and a 2‐(p‐toluidino)‐6‐naphthalene sulfonic acid assay. Incorporating another anionic ionizable lipid, oleic acid not only modulates the LNPs’ physicochemical properties, such as size, internal phase nanostructure, and surface charge but also synergistically enhances the antimicrobial potency together with ALC‐0315 with a benefit enhancing permeability and fusion with bacterial membranes. This study introduces a strategy for tailoring ionizable lipid compositions in LNPs, providing a new approach to antimicrobial treatment contributing to the fight against AMR.

## Graphical abstract

Lipid cubosomes comprising monoolein, ionizable cationic lipid ALC‐0315, and ionizable anionic oleic acid are developed as an antimicrobial drug delivery system. The synergistic effect of the internal inverse cubic structure and the ionic lipid molecular interactions significantly enhances the membrane permeability of piperacillin‐loaded particles, leading to increased Gram‐negative bacteria growth inhibition.

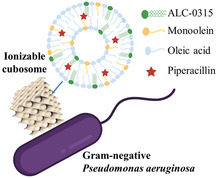

## 1 Introduction

The rise of antimicrobial resistance (AMR) has emerged as a formidable challenge in the global health landscape.^[^ ^1^, ^2^, ^3^ ^]^ Often referred to as the “silent pandemic”, AMR threatens to undermine the foundation of modern healthcare. Historically, antibiotics have been the cornerstone of bacterial infection treatments. However, their effectiveness is increasingly being challenged by the intricate defense mechanisms of bacteria. Gram‐negative bacteria, in particular, possess selective membranes that act as formidable barriers against many antimicrobial agents. Their defense is further bolstered by mechanisms like efflux pumps that actively expel harmful agents.^[^ ^4^, ^5^ ^]^ The extensive and indiscriminate use of antibiotics has accelerated the emergence of bacterial strains resistant to conventional treatments with predictions suggesting that AMR could be responsible for millions of deaths annually by 2050.^[^ ^6^, ^7^ ^]^

To combat this burgeoning crisis, nanoparticle‐based therapeutic delivery is emerging as an effective and safe means of delivering antimicrobial drugs to infection sites.^[^ ^8^, ^9^, ^10^ ^]^ Due to the biocompatible and biomimetic nature of lipids, self‐assembled lyotropic liquid crystalline lipid nanoparticles (LNPs) with membrane fusogenic properties have demonstrated broad‐ranging potential as promising nanocarriers in drug delivery applications.^[^ ^11^, ^12^, ^13^ ^]^ Lyotropic liquid crystalline LNPs with an inverse bicontinuous cubic phase (cubosomes) or an inverse hexagonal phase (hexosomes) have drawn significant interest because of their stability, high biocompatibility, high loading capacity, and sustained drug release properties.^[^ ^14^, ^15^, ^16^ ^]^ The bicontinuous cubic structure of cubosomes provides a large surface area, which can accommodate various antimicrobial agents, such as melittin, indolicidin, and gramicidin, with a high encapsulation efficiency of over 85%.^[^ ^17^ ^]^ Additionally, the membrane fusion mechanism of the cubic phase is significant, especially for the effective passage of biological membrane barriers such as the Gram‐negative bacterial outer plasma membrane, where uptake mechanisms are heavily restricted.^[^ ^18^, ^19^, ^20^ ^]^ This fusion mechanism can facilitate efficient drug uptake by the pathogens and enhance the antimicrobial delivery efficacy.

**Figure 1:**
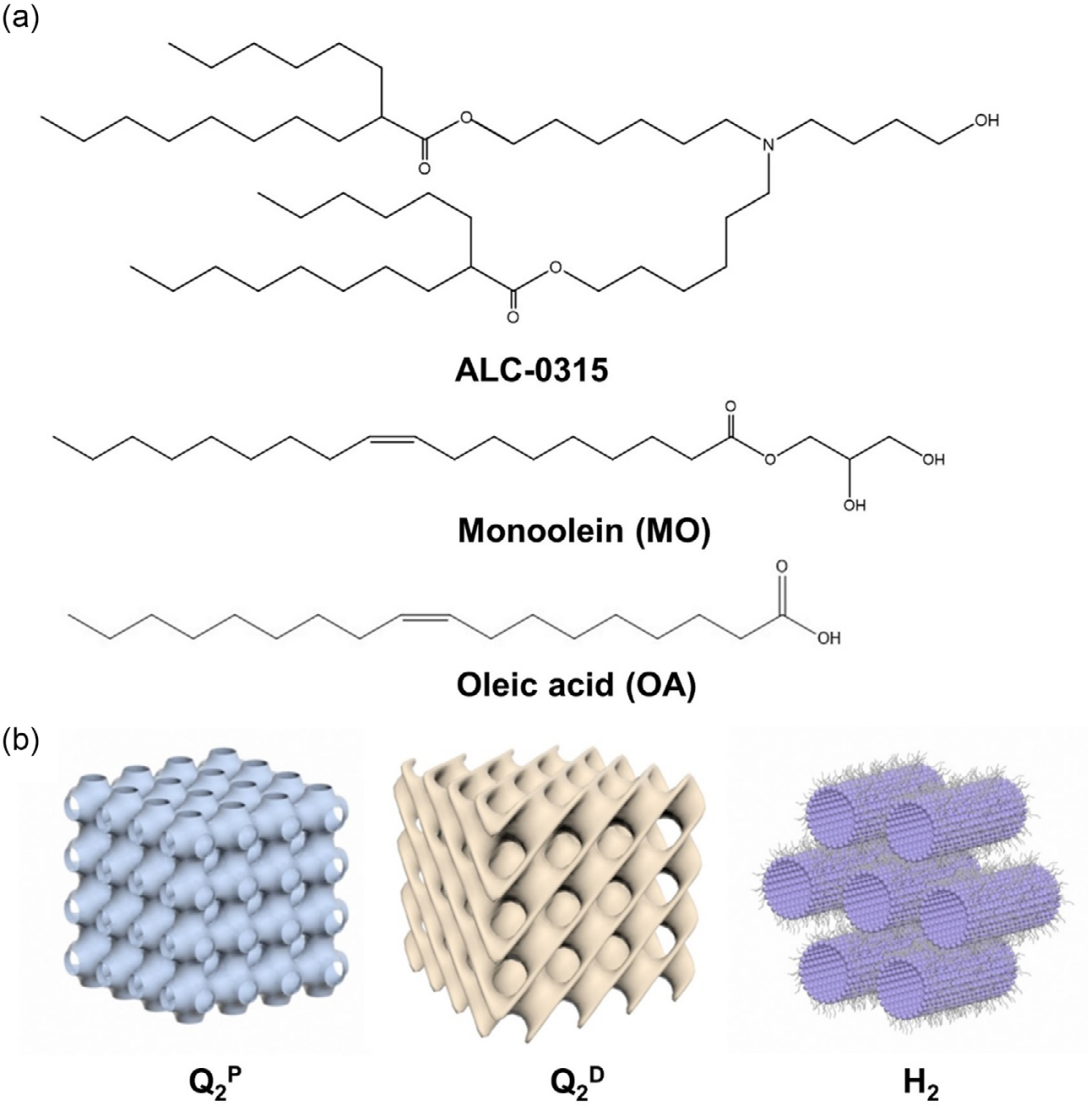
a) Molecular structures of ionizable lipid ALC‐0315, monoolein (MO), and oleic acid (OA). b) Schematic drawings of the inverse primitive cubic phase with the Im3m space group (Q_2_^P^), the inverse diamond cubic phase with the Pn3m space group (Q_2_^D^), and the inverse hexagonal (H_2_) phases.

Recently, LNPs have played a crucial role as nanocarriers in the context of mRNA‐based vaccines for COVID‐19. A key component of these vaccine LNPs is a cationic ionizable lipid, which plays a pivotal role in encapsulating and delivering the nucleic acid payload into cells.^[^ ^21^, ^22^ ^]^ The unique ionizable nature of the ionizable lipid, which can adjust molecular charge based on the surrounding pH, offers a strategic advantage in maintaining a neutral state for safety in neutral pH conditions while acquiring a positive charge in acidic conditions for cargo stabilization and endosomal escape.^[^ ^23^, ^24^, ^25^ ^]^ Such properties not only minimize potential toxicity in the bloodstream but also potentially enhance targeted drug delivery, especially in environments like bacterial infections where low pH environments are commonplace.^[^ ^26^ ^]^ Of note, ALC‐0315 (molecular structure in **Figure** **1**a) is a cationic ionizable lipid, containing a tertiary amine group that becomes positively charged in acidic environments, used in the mRNA BNT162b2 SARS‐CoV‐2 vaccines from BioNTech/Pfizer.^[^ ^27^ ^]^

In the context of antimicrobial therapy, Piperacillin (Pip), a broad‐spectrum antibiotic, is widely used to combat bacterial infections.^[^ ^28^, ^29^, ^30^, ^31^ ^]^ However, challenges related to its solubility, bioavailability, and targeting delivery necessitate the exploration of novel delivery systems to optimize its therapeutic potential. To address these challenges, this work explores the incorporation of a cationic ionizable lipid (ALC‐0315) and an anionic fatty acid into monoolein (MO)‐based LNPs as potential nanocarriers for Pip delivery. MO, also known as glyceryl monooleate, is a glycerol‐based lipid where a single oleic acid (OA) molecule is esterified to glycerol, as shown in Figure 1a. MO is a well‐known lipid used for cubosome formation stabilized by the copolymer Pluronics F127 (F127),^[^ ^14^, ^32^ ^]^ exhibiting an inverse primitive cubic phase with the Im3m space group (Q_2_^P^, as demonstrated in Figure 1b). The ionizable nature and the molecular structure of ALC‐0315 are proposed to enable LNPs to display inverse mesophases and respond to pH changes, particularly in the acidic environments typically associated with bacterial infections within the body. Additionally, OA, an anionic ionizable fatty acid (Figure 1a), was also incorporated, which contains a carboxylic acid group that deprotonates at relatively high pH conditions. OA has been used as a multifunctional pharmaceutical component in enhancing the permeability and stability of lipid‐based formulations.^[^ ^33^, ^34^, ^35^ ^]^ We have systematically studied the mesophase structures, physicochemical properties, and ionization behavior of MO‐based LNPs with the incorporation of both ALC‐0315 and OA, using synchrotron small angle X‐ray scattering (SAXS), cryogenic transmission electron microscopy (cryo‐TEM), dynamic light scattering (DLS), and a 2‐(p‐toluidino)‐6‐naphthalene sulfonic acid (TNS) assay. Finally, the Pip‐loaded cubosomes were evaluated for antimicrobial activity against a critical antibiotic‐resistant Gram‐negative strain, *Pseudomonas aeruginosa* (*P. aeruginosa*). Our results show that the incorporation of the ionizable species in the LNP formulations can enhance the antimicrobial efficacy of Pip, opening up new application potential for the clinically successful ionizable LNPs.

## 2 Experimental Section

### 2.1 Materials

Monoolein (MO, >99%) was purchased from Nu‐chek‐Prep Inc. (Elysian, MN, USA). (4‐hydroxybutyl) azanediyl)bis (hexane‐6,1‐diyl)bis(2‐hexyldecanoate) (ALC‐0315) was purchased from Advanced Molecular Technologies (Scoresby, Victoria, Australia). F127, oleic acid (OA), 2‐(p‐toluidino)‐6‐naphthalene sulfonic acid (TNS), citrate acid (≥99.5%), sodium citrate monobasic (≥99.5%), sodium phosphate dibasic (≥99.0%), sodium phosphate monobasic (≥99.0%), sodium bicarbonate (≥99.7%), sodium carbonate (≥99.5%), hydrochloric acid (HCl, 37%), sodium hydroxide (NaOH, ≥97.0%), nitric acid (HNO_3_), potassium hydroxide (KOH, ≥85%), potassium nitrate (KNO_3_, ≥99.0%), dimethyl sulfoxide (DMSO), and Pip were purchased from Sigma‐Aldrich (MI, USA). Methanol (analytical reagent) was obtained from Univar Ajax Finechem (NSW, Australia). All chemicals were used without further purification.

### 2.2 LNP Formulation Using Thin‐Film Homogenization Method

MO (50 mg), ionizable lipid (ALC‐0315), and OA were first dissolved in methanol, added to a 1.5 mL Eppendorf tube, and dried in a vacuum oven at 40 °C overnight, following the specific formulations in **Table** **1**. After drying, the mixture was homogenized in 1 mL F127 aqueous solution (10 wt% relative to MO) using a probe sonicator (Branson ultrasonifier 250, 35% Duty Cycle, 3 s on/5 s off, 5 min). For antimicrobial drug‐loaded samples, Pip (3 mol% relative to MO) was dissolved in ethanol and added to the dissolved lipid mixture before drying.

**Table 1:** Formulation of all LNPs.

Sample name	MO [mg mL^−1^]	ALC‐0315 (mol% to MO)	OA (mol% to MO)
MO	50	0	0
MO–1%ALC	50	1	0
MO–2%ALC	50	2	0
MO–5%ALC	50	5	0
MO–10%ALC	50	10	0
MO–5%ALC–1%OA	50	5	1
MO–5%ALC–2%OA	50	5	2
MO–5%ALC–5%OA	50	5	5

### 2.3 LNP Characterization

LNP characterization, including DLS and zeta potential measurements, SAXS, cryo‐TEM, and TNS assay were performed following the procedures reported in our recent work.^[^ ^25^, ^36^, ^37^ ^]^ The details of experimental methods are described in the supporting information. pH values of all LNP samples (diluted 10× with Milli‐Q water) after preparation were tested using a Mettler Toledo bench‐top pH meter.

### 2.4 Bacterial Culture and Antimicrobial Activity Testing

An MTS assay kit (Thermo Fisher) was employed to assess cell viability, utilizing the mean activity of mitochondrial succinate dehydrogenase as an indicator. *Pseudomonas aeruginosa* ATCC 2835 bacterial cells were cultured in nutrient broth medium within 10 mL sterile plastic screw cap centrifuge tubes, maintaining an optical density (OD600) range of 0.5–0.6, and cultivated at 37 °C with agitation at 150 rpm.

The cell viability determination assay for the *P. aeruginosa* ATCC 2835 bacterial strain was conducted for all samples, including free Pip, Pip@LNPs, and LNPs at the desired concentrations. A 1% bacterial inoculum was added to the positive control (free Pip in DMSO) and the negative controls (i.e., untreated bacteria culture) at the desired concentrations. Various concentrations of Pip@LNPs and LNPs were filtered using a sterile millex‐GP syringe filter (0.45 μm, PES, Millipore) before being utilized in the experiments. Free Pip (5 mg mL^−1^ in DMSO), Pip@LNPs, and LNPs with desired concentrations were added at the time of inoculation to 5 mL of culture medium (with a 1% inoculum) and incubated for 20–24 h, during which bacterial growth reached the stationary phase at 37 °C with agitation at 150 rpm. pH values of untreated and sample‐treated bacterial culture medium were measured to be 7.0 and ≈7.2, respectively. Subsequently, a duplicate 100 μL of cell culture medium was extracted from each sample (with 3 biological repeats, *n* = 3) and dispensed into a 96‐well plate. Bacterial cell viability was assessed using the MTS assay kit. 10 μL of MTS solution was added per 100 μL of sample and incubated for 30 min at 37 °C. Absorbance at 490 nm was measured using a microplate reader (SpectraMax, Molecular Devices). The absorbance obtained from the untreated bacterial culture was designated as 100% cell viability, and all other sample data were adjusted accordingly relative to this value.

### 2.5 Statistical Analysis

Graphs and data analysis were performed using GraphPad Prism 10 (GraphPad Software Inc, San Diego, CA, USA). For *Z*_average_ size, polydispersity index (PDI), intensity mean, and zeta potential of samples, data were shown as mean ± standard deviation (SD), *n* = 3. The pKa^app^ of LNPs and minimum inhibitory concentration of the antimicrobial that inhibits 50% of the bacterial growth (MIC50) were obtained by fitting the data using the Absolute IC50 Model in GraphPad Prism Software. A comparison of bacterial inhibition of samples at 50 μg mL^−1^ Pip concentration was determined by using an ordinary one‐way ANOVA test followed by Dunnett's multiple comparisons test. A *p*‐value of <0.05 was considered significant.

## 3 Results and Discussion

### 3.1 Physiochemical Properties of Ionizable ALC‐0315 Doped LNPs

**Figure 2:**
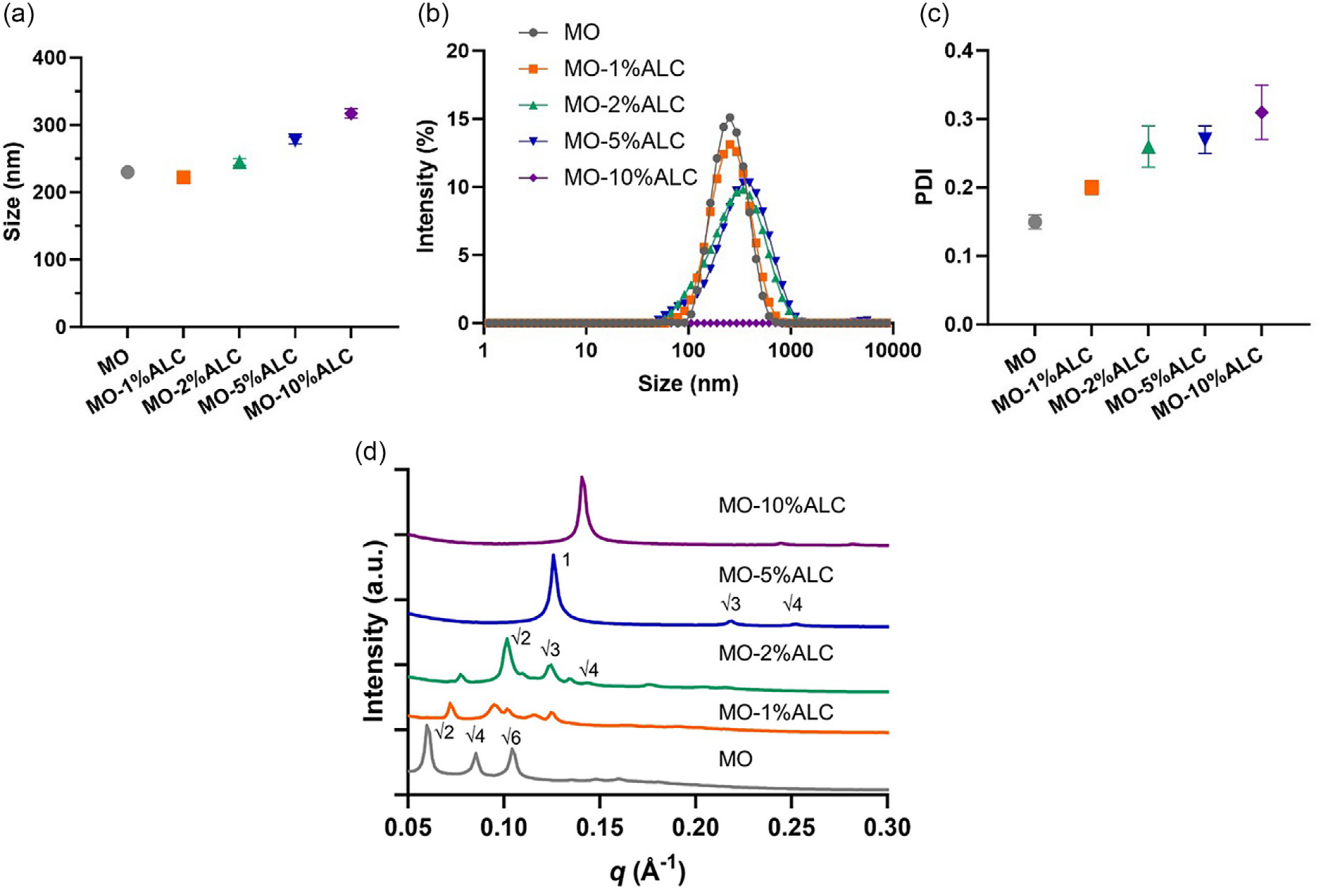
a) *Z*_average_ size, b) intensity profile, c) PDI, and d) 1D SAXS diffraction patterns of MO‐ALC LNPs with the molar concentrations of ALC‐0315 from 0 to 10% (relative to MO) prepared in water, measured at room temperature 25 °C, *n* = 3.

ALC‐0315 doped LNPs with different ALC‐0315 molar concentrations from 0 to 10% (relative to MO), stabilized with 10 wt% F127 (relative to MO) in water, were produced using a typical top‐down lipid dry film and sonication method.^[^ ^14^, ^32^ ^]^ The *Z*_average_ size, intensity profiles, and PDI were determined by DLS measurements and shown in **Figure** **2**a–c and Table S1, Supporting Information. The particle size of ALC‐0315 doped LNPs increased from 230 nm to over 300 nm (with the corresponding intensity mean increasing from 268 ± 98 to 363 ± 101 nm) and PDI increased from 0.15 to 0.31 as the molar concentration of the ALC‐0315 ionizable lipid increased. This size variation of LNPs influenced by the molar ratio of the ionizable lipid is consistent with our previous study on ALC‐0315/MO nanosystem and includes additional data points at molar ratios of 1% and 2%.^[^ ^25^ ^]^

The mesophase nanostructures for the LNPs were characterized using synchrotron SAXS and representative 1D SAXS plots of intensity versus scattering vector q for the nanoparticles are plotted in Figure 2d. The MO LNPs without ALC‐0315 demonstrated a typical Q_2_^P^ mesophase with the characteristic √2:√4:√6 peak spacing ratios in the diffraction pattern and a lattice parameter of 148.1 Å, which is consistent with previous work.^[^ ^38^ ^]^ As ALC‐0315 was doped into the system, MO‐1%ALC and MO‐2%ALC displayed a mixture of cubic Q_2_^P^ and the inverse diamond cubic phase with the Pn3m space group (Q_2_^D^, with √2:√3:√4 peak spacing ratio, as sketched in Figure 1b). For MO‐1%ALC, the lattice parameter of the Q_2_^P^ phase decreases to 123.4 Å compared with MO (148.1 Å) and the lattice parameter of the Q_2_^D^ phase is 93.5 Å. For MO‐2%ALC, the diffraction peaks of Q_2_^D^ with higher curvature are more obvious than those of Q_2_^P^. The lattice parameters of both Q_2_^P^ and Q_2_^D^ phases further decreased to 115.4 and 87.1 Å, respectively, also suggesting an increased membrane curvature as ALC‐0315 content was increased. The Bonnet ratio, namely, the ratio of the lattice parameters of coexisting Q_2_^P^/Q_2_^D^, was calculated to be 1.32 for both MO‐1%ALC and MO‐2%ALC, which is close to the theoretical value of 1.279.^[^ ^39^, ^40^ ^]^ As the ALC‐0315 concentration continued to increase, the inverse hexagonal (H_2_) phase (also sketched in Figure 1b) with the characteristic diffraction peaks at 1:√3:√4 spacing ratios was formed within the interior of the MO‐5%ALC and MO‐10%ALC samples and the cubic phases disappeared. While both samples showed the H_2_ phase, the diffraction peaks of the MO‐10%ALC sample were located at higher *q* values than the MO‐5%ALC sample, with the lattice parameter correspondingly decreasing from 57.3 Å for MO‐5%ALC to 51.2 Å for MO‐10%ALC.

**Figure 3:**
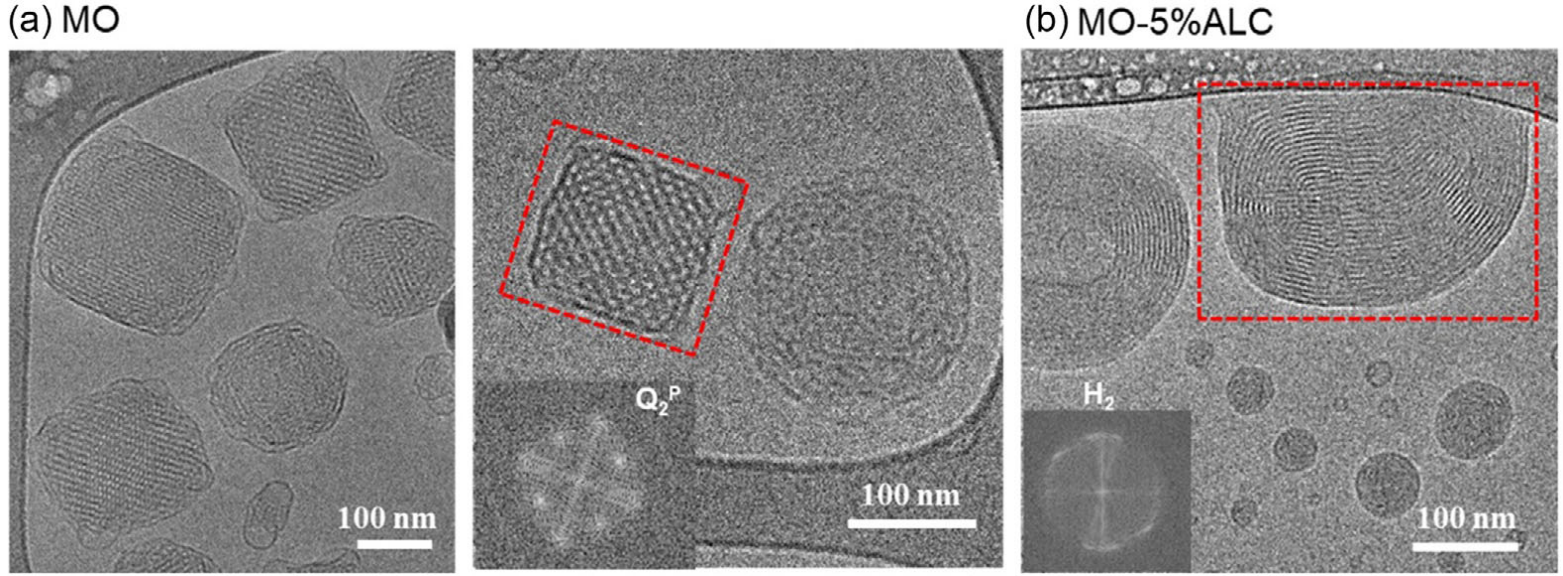
a) Cryo‐TEM images of MO and b) MO‐5%ALC, respectively. Scale bar: 100 nm. The insets show the FFT‐generated images of the periodic patterns observed in the red dashed box, revealing the Q_2_^P^ and H_2_ phases, respectively.

Cryo‐TEM was used to further characterize the internal structure of MO and MO‐5%ALC LNPs, and representative images are shown in **Figure** **3**a.b. Cryo‐TEM imaging of MO LNPs (Figure 3a) revealed a nanoparticle size of about 200 nm and LNPs adopted a characteristic petal‐like morphology with membrane blebs encompassing a highly ordered inner core, coexisting with vesicles.^[^ ^41^ ^]^ Fast Fourier transform (FFT) analysis showed a two‐dimensional cubic symmetry, suggesting the reflections from {110} and {200} planes of the cubic Q_2_^P^ phase with a measured lattice parameter of 152.5 Å which is close to the SAXS result (Figure 2c).^[^ ^42^, ^43^ ^]^ For the cryo‐TEM image of MO‐5%ALC LNPs (Figure 3b), the typical “fingerprint” patterns were observed;FFT analysis of the H_2_ phase is consistent with a measured lattice parameter of 53.8 Å,^[^ ^44^ ^]^ which is close to the SAXS results in Figure 2c.

From the synchrotron SAXS and the cryo‐TEM results, it is clear that adding ALC‐0315 to the lipid system caused higher membrane curvature following the sequence of the Q_2_^P^ phase for the original F127‐stabilized MO cubosomes to Q_2_^D^ then to H_2_. In the realm of mixed lipid self‐assembly systems, mesophase transitions are generally explained by using the critical packing parameter (CPP) concept defined in Equation (1)^[^ ^45^ ^]^

(1) $$\text{CPP}=\frac{v}{a\times l}$$

where *v* represents the effective volume of the hydrophobic tail(s), *a* is the effective hydrophilic headgroup area, and *l* is the effective length of the hydrophobic chain/s.^[^ ^15^, ^46^ ^]^ With a 4‐carbon chain tail structure (i.e., large *v*), the effective CPP value of the ALC‐0315 molecule in a neutral environment is calculated to be 1.33,^[^ ^36^ ^]^ higher than MO's CPP value of 1.17.^[^ ^47^ ^]^ When mixed in the system, the effective CPP value increased with higher concentrations of ALC‐0315, pushing the system into higher membrane curvatures. In our previous work, a 5% ALC‐0315 doping led to a direct mesophase transition to the H_2_ phase. In this work, the observation of mixed cubic phases of the Q_2_^P^ and Q_2_^D^ in the MO‐1%ALC and MO‐2%ALC was as expected based on the effective CPP consideration. Based on the consideration of good monodispersity (with PDI less than 0.3) of samples and preferable cationic fusogenic component interacting with bacteria,^[^ ^48^ ^]^ we selected MO‐5%ALC as the benchmark for the subsequent studies in this work.

### 3.2 OA‐Incorporated Ionizable LNPs

**Figure 4:**
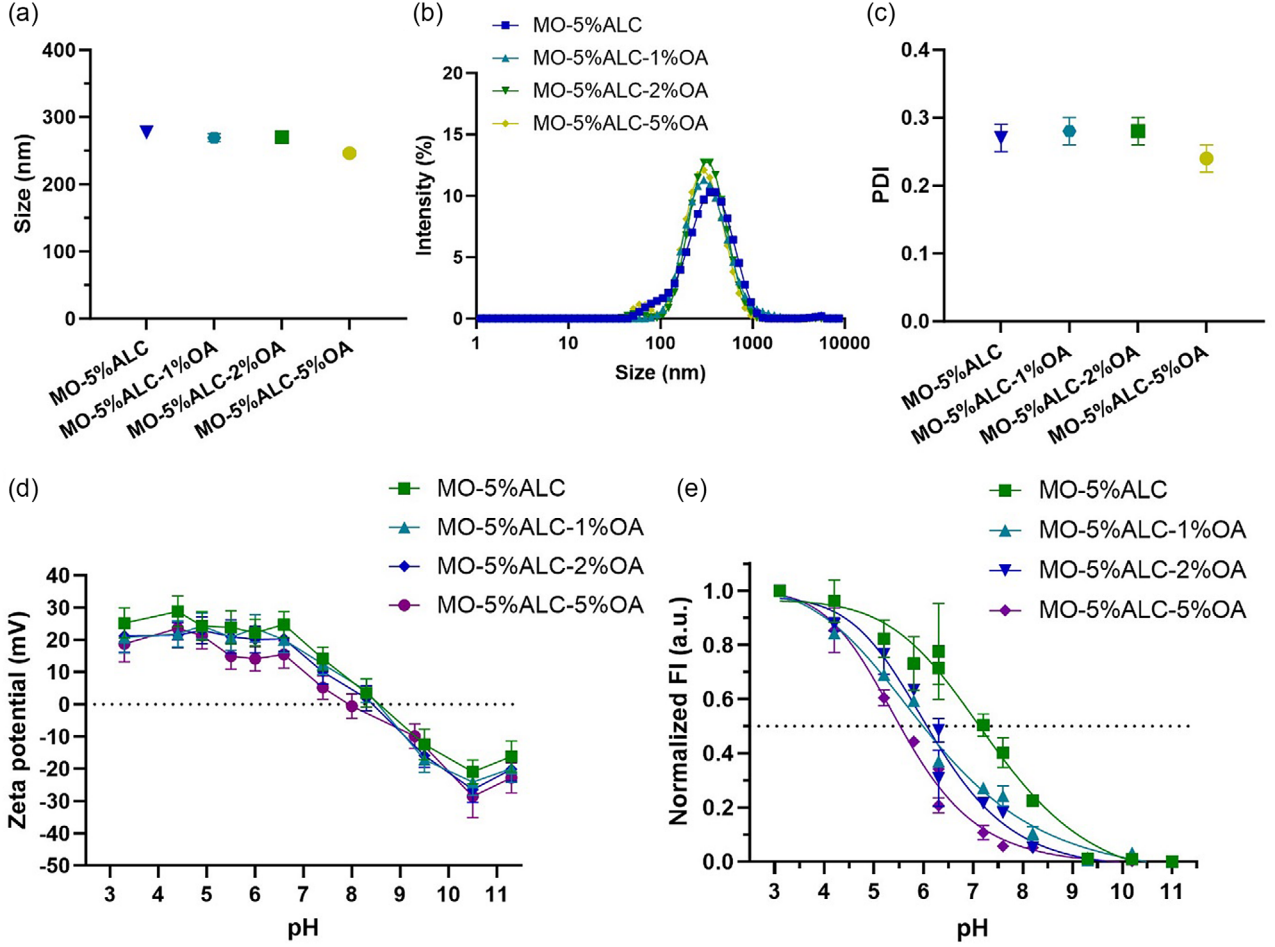
a) *Z*_average_ size, b) intensity profile, c) PDI, and d) pH‐dependent zeta potential of the OA‐incorporated MO‐5%ALC LNPs with OA concentrations at 1%, 2%, and 5%. e) Normalized cationic surface charge of OA‐incorporated MO‐5%ALC LNPs measured using TNS assay.

pH‐responsive nanoparticles show promise for delivering therapeutic cargos, e.g., antimicrobial peptides, precisely to infection areas. The sites of bacterial infection areas typically exhibit reduced pH values. Considering the pH variation at various infection locations, such as pH ≈ 5.6 within *P. aeruginosa* biofilms,^[^ ^49^ ^]^ pH down to 3.9 at the plaques of dental caries,^[^ ^50^ ^]^ and pH ≈ 5.5–6.7 at infected wounds,^[^ ^51^ ^]^ there is a need for developing LNPs that can adapt to the specific pH at the local pathological conditions.^[^ ^26^ ^]^ To further modulate the physiochemical properties of the ionizable LNPs, we incorporated an unsaturated fatty acid, OA with a long C18 chain containing an unsaturated bond between C9 and C10 with the *cis* configuration, as a pH‐responsive anionic component into the LNP formulation. According to **Figure** **4**a–c and Table S2, Supporting Information, the incorporation of OA at 1%, 2%, and 5% (an equal molar concentration to the LNP sample containing 5% ALC‐0315) resulted in a gradual *Z*_average_ size decrease from 277 to 246 nm, an intensity mean size decrease from 373 ± 201 to 326 ± 138 nm, and a small effect on PDI, demonstrating the component effect on the particle size.

All LNPs show a pH‐dependent behavior of the surface charge (Figure 4d), with positive zeta potential values at acidic conditions (pH < 7) and negative zeta potential values at basic conditions (pH > 8). This pH‐dependent surface charge behavior of the LNPs reflects the protonation and deprotonation of the amino headgroup of the ALC‐0315 and the carboxyl group of OA and demonstrates the responsive nature of these nanocarriers, with a near‐neutral charge around physiological pH for administration but a more interactive and positive surface charge for specific pathological environments, e.g., bacterial infections. MO‐5%ALC‐5%OA with the maximum OA amount has a lower zeta potential value (≈10 mV) than others at a pH range of 5–8, which can be attributed to the negatively charged oleate ions.^[^ ^52^, ^53^ ^]^

ALC‐0315 behaves as a weak base with an intrinsic pK_a_ (pKa^0^) value of ≈7.8 at a self‐assembled lipid‐water interface.^[^ ^36^, ^37^ ^]^ The population of ionized positively charged lipids increases as the pH decreases over a certain pH range. OA behaves as a weak acid with a pKa^0^ value of ≈6.7.^[^ ^54^ ^]^ The population of ionized negatively charged lipids increases as the pH increases over a certain pH range. As a consequence, in the system that possesses both ALC‐0315 and OA, there is certainly a competing acid‐base equilibrium in certain pH ranges. The overall surface potential will be governed by the ionization of a prototropic moiety residing at a lipid‐aqueous solution interface. This can be described in terms of the impact of the interfacial microenvironment on the acid–base equilibria with ${\text{pK}}_{a}^{\text{app}}={\text{pK}}_{a}^{0}-\frac{e\Psi }{2.303kT}$, where pKa^app^ is the apparent pKa of the ionizable lipid, pK_a_^0^ is the intrinsic pK_a_ in the absence of charge at the interface, *e* is the elementary electrostatic charge, Ψ is the mean‐field surface potential originating from the surface charge, *k* is the Boltzmann constant, and *T* is the absolute temperature.^[^ ^54^, ^55^ ^]^ When there are two ionizable lipids with close pK_a_^0^ values (e.g., ALC‐0315 with ≈7.8 and OA with ≈6.7) the ratio of cationic lipid to anionic lipid at a particular pH will be an important consideration influencing the overall surface charge density at the self‐assembled lipid‐aqueous solution interface.

The ionization of the cationic lipid was further characterized using the TNS assay (Figure 4e). Results show the inclusion of OA as a pH‐responsive anionic component in the LNPs efficiently mediated the ionization of the LNPs. The pH in the TNS assay where the protonated and deprotonated form of the cationic ionizable lipid existing at equal concentrations is considered as the apparent pKa of LNPs at the 50% ionization point pH. This value reduced from 7.2 (MO‐5%ALC) to 5.9 (MO‐5%ALC‐1%OA), 6.1 (MO‐5%ALC‐2%OA), and 5.5 (MO‐5%ALC‐5%OA). This apparent pKa decrease can be attributed to the linked performance of both the pH‐responsive amino group in ALC‐0315 and the carboxyl group in OA as a cationic–anionic pair. This finding reveals the feasibility of mediating LNPs’ apparent pKa via incorporating additional anionic components, e.g., ionizable fatty acids, for selective pathology‐specific pH‐triggered antimicrobial delivery.^[^ ^25^, ^26^ ^]^ It is worth noting that the apparent pKa varies as a function of pH.

**Figure 5:**
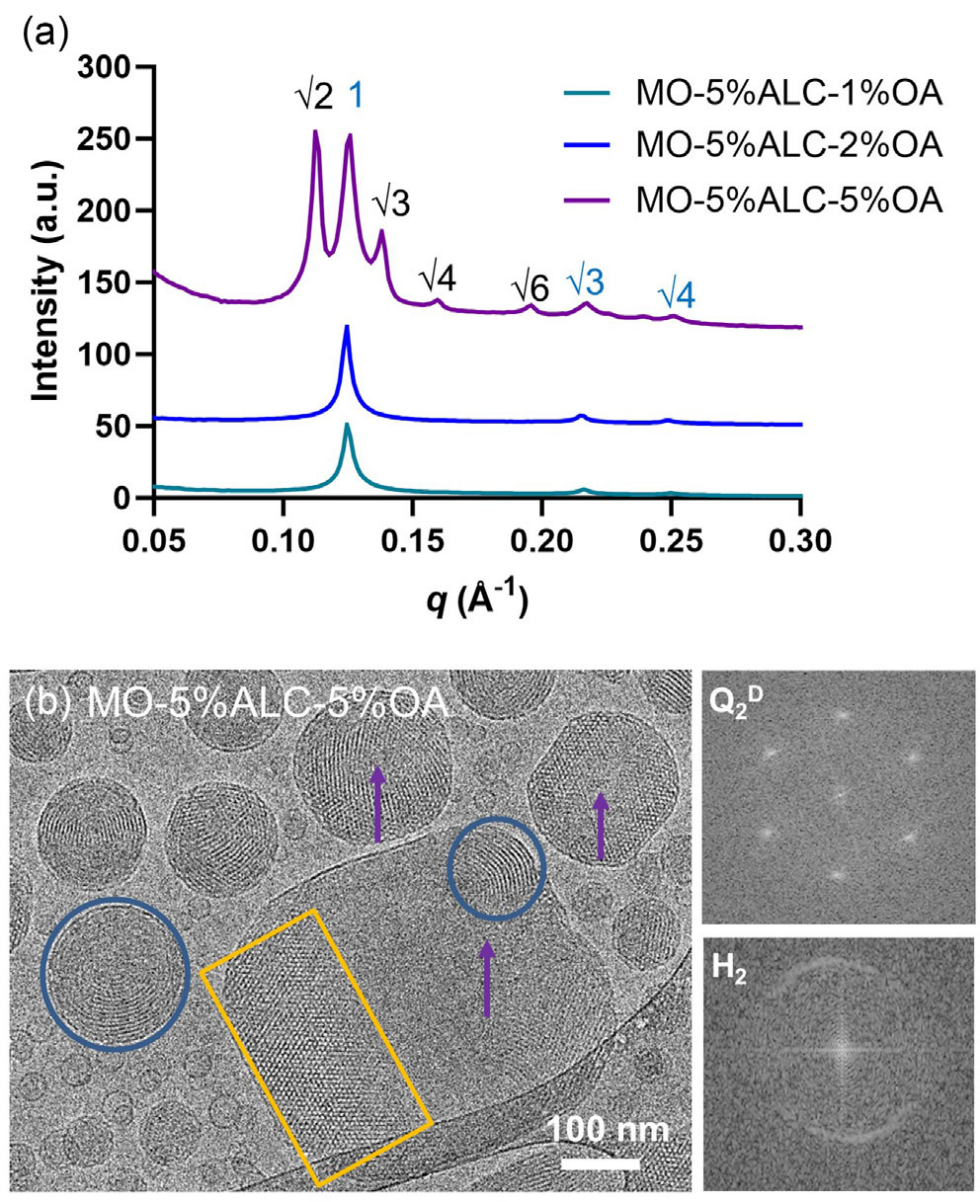
a) SAXS diffraction patterns of OA‐incorporated MO‐5%ALC LNPs with the molar concentrations (1, 2, and 5%) of OA in water at room temperature 25 °C. b) Cryo‐TEM image of MO‐5%ALC‐5%OA LNPs. Representative regions for the Q_2_^D^ phase are indicated by a yellow square, the H_2_ phase is indicated by blue circles, and the sponge phase existing in the center of the particles is indicated by purple arrows. The corresponding FFT images of the Q_2_^D^ and H_2_ phases are presented.

The structural effect of OA incorporation into the MO‐5%ALC LNPs was subsequently characterized by SAXS and cryo‐TEM, shown in **Figure** **5**. With 1% and 2% OA incorporation, MO‐5%ALC‐1%OA and MO‐5%ALC‐2%OA maintained the H_2_ phase with characteristic peaks at 1:√3:√4 peak spacing ratios, consistent with that of MO‐5%ALC in Figure 2c. Their lattice parameters (both 57.8 Å) are slightly increased compared to MO‐5%ALC (57.3 Å). As the amount of OA increased to 5%, a mixture of Q_2_^D^ and H_2_ mesophases was observed with the appearance of the Q_2_^D^ characteristic peaks (√2:√3:√4:√6). The lattice parameters of Q_2_^D^ and H_2_ mesophases are calculated to be 79.0 and 57.3 Å, respectively. The adopted Q_2_^D^ and H_2_ phases of MO‐5%ALC‐5%OA were further confirmed by cryo‐TEM, as shown in Figure 4b. FFT analysis of the internal long‐range ordered structures, indicated by yellow squares (Q_2_^D^) and blue circles (H_2_) in Figure 5b, further confirms the inverse structures^[^ ^41^, ^42^, ^43^, ^44^ ^]^ with a lattice parameter of 81.8 Å for Q_2_^D^ and 53.1 Å for H_2_, consistent with the previous SAXS results. Additionally, regions of sponge phase were observed, preferentially located close to the center of the particle (as indicated by purple arrows in some particles), which can be attributed to the local optimization of lipid monolayer curvature instead of an entropy increase.^[^ ^56^ ^]^ Interestingly, cryo‐TEM images revealed further details on the coexistence of Q_2_^D^, H_2_, and sponge phases within a single particle entity. These observed multiphase, or so‐called Janus LNPs could potentially work as smart nanocarriers with varying sustained release of bioactive cargos.^[^ ^42^ ^]^

Taken together, both the increase of lattice parameter and the partial phase transition from H_2_ to Q_2_^P^/sponge phase demonstrated the attenuation of membrane curvature with OA incorporation and indicated smaller effective CPP values. This is contradictory to the mesophase behavior of adding OA into MO‐based cubosomes without a different type of ionizable lipid, as reported in several articles.^[^ ^44^, ^57^ ^]^ OA is a molecule with a small headgroup and a large volume‐occupying hydrophobic tail. Therefore, it is known to increase the curvature in the self‐assembled lyotropic liquid crystalline mesophase containing LNPs. For example, Salentinig et al.^[^ ^57^ ^]^ showed that the membrane curvature increased within particles transitioning from the Q_2_ phase to the H_2_ and micellar cubic (I_2_) phases as the concentration of OA in MO‐based LNPs correspondingly increased from 0–20% to 20–60% and 60–90% in PBS buffer (pH 6.5). The distinct difference between the known behavior of OA and the opposite trend observed in this study can be attributed to the electrostatic interaction between the negatively charged carboxylate group of OA and the positively charged ammonium group of ALC‐0315. In this case, the pairing of charged functional groups of ALC‐0315 and OA could increase the effective hydrophilic surface area (*a*) in Equation (1) and thus the CPP value decreased compared to those of ALC‐0315‐loaded MO and OA‐loaded MO nanoparticles. This CPP decrease resulting from the charged ALC‐0315 and OA pair in LNPs is consistent with the CPP decrease driven by ALC‐0315 protonation^[^ ^25^, ^36^ ^]^ and OA deprotonation.^[^ ^57^ ^]^ This electrostatic attraction of head groups with opposite charges in LNPs is similar to what has been reported for catanionic surfactants, which consist of equimolar mixtures of anionic and cationic surfactants. They behave as synergistic systems analogous to zwitterionic double‐chain surfactants.^[^ ^58^, ^59^, ^60^ ^]^

### 3.3 Pip‐Loaded Ionizable Cubosomes

**Figure 6:**
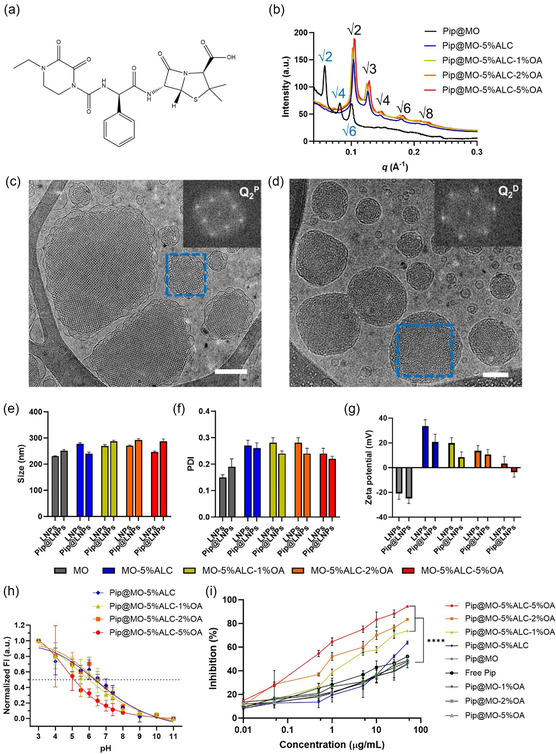
a) Molecular structure of piperacillin (Pip). b) SAXS diffraction patterns of Pip@LNPs. c,d) Cryo‐TEM images of (c) Pip@MO and d) Pip@MO‐5%ALC‐5%OA. Representative ordered regions for the cubic phases are indicated by the blue squares and the corresponding FFT images of the Q_2_^P^ and Q_2_^D^ phases are presented. Scale bar: 100 nm. e) Size, f) PDI, and g) Zeta potential in water of LNPs and Pip@LNPs, *n* = 3. h) Normalized cationic surface charge of Pip@LNPs measured using TNS assay, *n* = 3. i) Antimicrobial activity (inhibition, %) of Pip‐loaded cubosomes for Gram‐negative *P. aeruginosa* after 24 h incubation at 37 °C, *n* = 3. A comparison of bacterial inhibition of samples (Pip@MO‐5%ALC‐OA samples vs free Pip) at 50 μg mL Pip concentration was determined using an ordinary one‐way ANOVA test followed by Dunnett's multiple comparisons test, ****p < 0.0001.

Lipid nanocarriers can enhance the therapeutic effects of antimicrobial drugs by improving their bioavailability and solubility.^[^ ^61^, ^62^, ^63^ ^]^ They can ensure targeted delivery to specific sites and provide sustained drug release, which can reduce side effects and increase drug efficacy. Additionally, encapsulation in nanocarriers can protect healthy tissues from exposure to drug toxicity.^[^ ^64^, ^65^, ^66^ ^]^ OA‐incorporated ionizable LNPs were used as nanocarriers for a model antimicrobial cargo, piperacillin (Pip, **Figure** **6**a). Pip is a broad‐spectrum antibiotic extensively utilized in antimicrobial therapy to treat a variety of bacterial infections.^[^ ^28^, ^29^, ^30^, ^31^ ^]^ Based on our previous study, the optimized loading amount of Pip in MO cubosomes is selected as 3 mol%, with the consideration of less adverse structural effect from Pip cargo and inhibition against *P. aeruginosa*.^[^ ^67^ ^]^ The loading of 3 mol% Pip in the MO‐5%ALC LNPs which had a H_2_ phase resulted in a phase transition of the particle to Q_2_^D^ with characteristic √2:√3:√4 peak space ratios as seen in Figure 6b, which indicates a decrease of membrane curvature. In fact, in the ALC‐0315/MO/OA mixed LNP systems, Pip loading had a dominating effect on the internal mesophase showing consistent Q_2_^D^ phase with lattice parameters of 86.3 Å (Pip@MO‐5%ALC), 87.1 Å (Pip@MO‐5%ALC‐1%OA), 86.3 Å (Pip@MO‐5%ALC‐2%OA), and 85.4 Å (Pip@MO‐5%ALC‐5%OA). These results suggest that the loading of Pip into the LNPs dominated the molecular interactions within the self‐assembled object, stabilizing the Q_2_ phase over the H_2_ phase. This could be attributed to the interactions between various functional groups of Pip, such as amide groups, beta‐lactam ring, thiazolidine ring, and carboxyl groups, and the ionizable lipids ALC‐0315 and OA. For comparison, Pip@MO still maintained the primitive cubic Q_2_^P^ phase with a lattice parameter of 154.0 Å, which is larger than that of MO cubosomes without drug (≈140 Å).^[^ ^38^, ^68^ ^]^ And Pip@MO‐OA samples without ALC‐0315 also remained Q_2_^P^ phase with a decreasing lattice parameter from 144.9 to 125.8 Å as OA increases from 1% to 5%, as shown in Figure S3, Supporting Information.

Representative Pip‐loaded samples, Pip@MO and Pip@MO‐5%ALC‐5%OA, were further characterized using cryo‐TEM, as shown in Figure 6c,d. The long‐range ordered structure within the LNPs suggests the existence of inverse mesophases. The FFT analysis of highlighted areas by blue dashed lines confirms the Q_2_^P^ mesophase (with a lattice parameter of ≈147.7 Å) of Pip@MO and Q_2_^D^ mesophase (with a lattice parameter of ≈83.2 Å) of Pip@MO‐5%ALC‐5%OA, consistent with the SAXS data (Figure 6b). This cargo‐induced phase transformation demonstrates the necessity to consider the structural impact of therapeutic cargos when LNPs are used as drug delivery nanocarriers. Previous studies have shown that many cargos can cause mesophase shift in the interior of the LNPs, affecting their drug‐release behavior.^[^ ^69^, ^70^ ^]^

Moreover, all Pip@LNPs demonstrated a particle size of about 250 nm with a PDI in a range from 0.19 to 0.26, as shown in Figure 6e,f. The zeta potential and pH of the LNP samples were measured in water (Figure 6g and Table S2, Supporting Information). The loading of Pip cargo resulted in a decrease in zeta potential (over 4.0 mV) and pH values (over 1.0) of Pip@LNPs compared to the corresponding LNPs. The zeta potential value of Pip@MO is –25 ± 4 mV compared to the blank MO cubosomes (–21 ± 5 mV). MO‐5%ALC demonstrated a positive zeta potential value of 34 ± 5 mV with the inclusion of the ionizable lipid ALC‐0315. However, MO‐ALC‐OA samples showed a gradual decrease of the zeta‐potential values from 20 ± 4 mV to 3 ± 6 mV as the amount of OA, which is an anionic ionizable species, increased from 0% to 5%. In comparison, MO–OA samples without ALC‐0315 show negative zeta potential values of ≈–25 mV. An interesting observation is drug‐free MO‐5%ALC‐5%OA having a near neutral zeta potential value, indicating the presence of electrostatic interaction between the negatively charged OA and the positively charged ALC‐0315 molecules at an equal molar ratio in the measured system. Loading Pip caused various degrees of decrease in the zeta‐potential values. For example, loading Pip to MO‐5%ALC‐5%OA once again caused a slight decrease in the zeta potential value to −3.7 ± 4.0 mV of Pip@MO‐5%ALC‐5%OA. The cationic ionization behavior of Pip@LNPs was further investigated using TNS assay and shown in Figure 6h. The loading of Pip cargo also mediated the apparent pKa of Pip@LNPs, at the 50% ionization pH point, to be 6.5 (Pip@MO‐5%ALC), 5.9 (Pip@MO‐5%ALC‐1%OA), 6.5 (Pip@MO‐5%ALC‐2%OA), and 5.0 (Pip@MO‐5%ALC‐5%OA), compared to 7.2 (MO‐5%ALC), 5.9 (MO‐5%ALC‐1%OA), 6.1 (MO‐5%ALC‐2%OA), and 5.5 (MO‐5%ALC‐5%OA), correspondingly. A decrease of the apparent pKa value upon Pip loading in both MO‐5%ALC and MO‐5%ALC‐5%OA was observed, indicating that a lower pH condition is required to protonate 50% of the population of the ALC‐0315 lipid, which also suggests the interaction between lipid and Pip cargo.

The therapeutic effectiveness of the Pip@LNPs in inhibiting the growth of Gram‐negative bacteria—*P. aeruginosa* was evaluated, a critical antibiotic‐resistant strain with an extraordinary capacity for resistance through chromosomal mutations.^[^ ^71^, ^72^ ^]^ The growth inhibition of the bacteria after 24 h incubation with the Pip@LNPs at various antimicrobial drug (Pip) concentrations up to 50 μg mL^−1^ was evaluated and is shown in Figure 6f. The bacterial inhibition of free Pip drug dissolved in DMSO was tested as a positive control. All Pip@LNPs demonstrated a dose‐dependent inhibition effect on *P. aeruginosa*, reaching the most effective inhibition at the highest drug concentration, i.e., 50 μg mL^−1^ (Figure 6i). The bacterial inhibition of Pip‐free LNPs with the same lipid concentrations as the Pip‐loaded ones was also investigated for comparison and showed a maximum inhibition of ≈20% in Figure S2, Supporting Information, which demonstrates no significant inhibition. The inhibition effect at 50 μg mL^−1^ Pip concentration followed the order from high to low of Pip@MO‐5%ALC‐5%OA > Pip@MO‐5%ALC‐2%OA > Pip@MO‐5%ALC‐1%OA > Pip@MO‐5%ALC > Pip@MO ≈ Pip@MO‐(1, 2, 5)%OA ≈ free Pip (control). There is an obvious improvement in the inhibition effect observed after the inclusion of the ionizable cationic lipid (5% ALC‐0315) compared to free Pip and both Pip@MO and Pip@MO‐OA series. The best‐performing sample is clearly Pip@MO‐5%ALC‐5%OA achieving a maximum inhibition effect of 94 ± 1% at 50 μg mL^−1^ Pip concentration, which is approximately twice asy effective as those samples without ALC‐0315 with *p* < 0.0001. This reveals the important role of the cationic ionizable lipid, ALC‐0315, in the improvement of the inhibition effect, which could be attributed to the promoted fusogenic property with the outer bacterial membrane of *P. aeruginosa* and subsequent burst release of the antimicrobial drug.^[^ ^33^, ^48^, ^73^ ^]^

More interestingly, although Pip@MO‐5%ALC‐OA samples with 0, 1, 2, and 5% OA maintained a consistent Q_2_^D^ phase (Figure 6b) and underwent a surface potential decrease from positive to neutral charge, the inhibition obviously increased from 64 ± 1% to 94 ± 1% at 50 μg mL^−1^ Pip concentration. The value of MIC50 reduced from 2.8  μg mL^−1^ Pip concentration for Pip@MO‐5%ALC‐1%OA, 1.5 μg mL^−1^ for Pip@MO‐5%ALC‐2%OA, to 0.5 μg mL^−1^ for Pip@MO‐5%ALC‐5%OA. MIC50 values for the free Pip and the Pip@MO‐OA samples could not be determined by the IC50 model as the maximal inhibition at the highest drug concentration was only in the range of 40–50%. Considering the incorporation of OA in Pip@MO‐OA samples without ALC‐0315 did not increase the inhibition effect, this significant improvement of growth inhibition (*p* < 0.0001) between Pip@MO‐5%ALC‐OA samples and free Pip demonstrates the synergistic effect of OA and ALC‐0315. This highlights the synergistic role of ALC‐0315 and OA in enhancing the permeability of cubosomes and rapidly fusing with the membrane of *P. aeruginosa*. This finding is also consistent with the literature where Selvadoss et al.^[^ ^33^ ^]^ reported the enhancement of antimicrobial activity on 32 strains of *P. aeruginosa* by using OA‐incorporated liposomes to deliver six different classes of antibiotics. Overall, our developed Pip‐loaded cubosomes containing two pH‐responsive ionizable lipids have shown excellent antimicrobial efficacy and future studies can focus on target delivery of these particles tunable in terms of mesophase structure, surface charge, and ionization behavior to infection sites of various pH and physiological conditions.^[^ ^62^, ^63^, ^66^, ^74^ ^]^ It is also worthwhile to mention that cubosome nanocarriers can work as a versatile platform for codelivering multiple antimicrobials to achieve potential synergistic effects against resistant bacterial strains, e.g., the combination of Pip and tazobactam.^[^ ^17^, ^28^, ^75^ ^]^

## 4 Conclusion

In summary, we explored the intricate interplay between the lyotropic liquid crystalline structure‐forming lipid MO, the cationic ionizable lipid ALC‐0315, and the anionic ionizable fatty acid OA to tailor the physiochemical properties of LNPs for antimicrobial drug delivery. The incorporation of ALC‐0315 and OA demonstrated a profound influence on many aspects of the LNPs, presenting a versatile platform with tunable properties including inverse mesophases known to promote membrane fusion, surface charge, and apparent pKa (ionization) behavior. As more ALC‐0315 was doped into LNPs from 0 to 10%, the membrane curvature of LNPs increased, transforming from the Q_2_ phases to the H_2_ phase. Moreover, the incorporation of OA into the MO‐5%ALC LNPs mediated the size, pH‐dependent surface charge, and apparent pKa of ALC‐0315 at 50% ionization pH point (from 7.2 to 5.5) of LNPs. Both SAXS and cryo‐TEM characterization of the LNPs revealed an increase in the lattice parameters and a phase transition from the H_2_ phase to the Q_2_ phase as OA content increased from 0 to 5%. Importantly, our findings diverged from previous reports suggesting an increase in the membrane curvature with the incorporation of either ALC‐0315 or OA into cubosomes, showcasing a unique electrostatic interaction between the charged functional groups of ALC‐0315 and OA, which resulted in a decrease in membrane curvature.

The encapsulation of Pip within LNPs led to Q_2_^D^ phase formation and demonstrated drug‐induced mesophase transition of LNPs. The observed dose‐dependent inhibition effect on *P. aeruginosa* reveals that, at the fixed 5% molar ratio of ALC‐0315 in LNPs, increased OA concentrations correlate with enhanced bacterial inhibition, underscoring the synergistic role of ALC‐0315 and OA in increasing cubosome permeability and facilitating their fusion with bacterial membranes. Our study presents an innovative approach to customize ionizable LNPs for effective antimicrobial drug delivery. By strategically combining ALC‐0315 and OA, we introduce a versatile platform with potential in pathology‐specific pH‐triggered antimicrobial delivery beyond conventional drug delivery systems. This antibiotic‐loaded cubosome formulation emerges as a promising therapeutic strategy to address the growing challenge of multidrug‐resistant *P. aeruginosa*.

## Conflict of Interest

The authors declare no conflict of interest.

## Supporting information

Supplementary Material

## Data Availability

The data that support the findings of this study are available from the corresponding author upon reasonable request.

## References

[1] World Health Organization , Global Priority List of Antibiotic‐Resistant Bacteria to Guide Research, Discovery, and Development of New Antibiotics, World Health Organization, Geneva, Switzerland 2017.

[2] A. Luther , M. Urfer , M. Zahn , M. Müller , S.‐Y. Wang , M. Mondal , A. Vitale , J.‐B. Hartmann , T. Sharpe , F. Lo Monte , H. Kocherla , E. Cline , G. Pessi , P. Rath , S. M. Modaresi , P. Chiquet , S. Stiegeler , C. Verbree , T. Remus , M. Schmitt , C. Kolopp , M.‐A. Westwood , N. Desjonquères , E. Brabet , S. Hell , K. LePoupon , A. Vermeulen , R. Jaisson , V. Rithié , G. Upert , et al., Nature 2019, 576, 452. 31645764 10.1038/s41586-019-1665-6

[3] A. R. Mahoney , M. M. Safaee , W. M. Wuest , A. L. Furst , iScience 2021, 24, 102304. 33748695 10.1016/j.isci.2021.102304 PMC7955580

[4] L. L. Silver , Bioorg. Med. Chem. 2016, 24, 6379. 27381365 10.1016/j.bmc.2016.06.044

[5] P. Cardoso , H. Glossop , T. G. Meikle , A. Aburto‐Medina , C. E. Conn , V. Sarojini , C. Valery , Biophys. Rev. 2021, 13, 35. 33495702 10.1007/s12551-021-00784-y PMC7817352

[6] World Health Organization , Global Excess Deaths Associated with COVID‐19 (Modelled Estimates), World Health Organization Geneve, Switzerland 2021.

[7] World Health Organization , Antimicrobial Resistance: Global Report on Surveillance, World Health Organization, Geneve, Switzerland, 2014.

[8] K. Bin Liew , A. K. Janakiraman , R. Sundarapandian , S. H. Khalid , F. A. Razzaq , L. C. Ming , A. Khan , A. Kalusalingam , P. W. Ng , J. Med. Life 2022, 2022, 328. 10.25122/jml-2021-0097 PMC9015166 35449993

[9] K. Forier , K. Raemdonck , S. C. De Smedt , J. Demeester , T. Coenye , K. Braeckmans , J. Controlled Release 2014, 190, 607. 10.1016/j.jconrel.2014.03.055 24794896

[10] Y. Deng , R. Huang , S. Huang , M. Xiong , BIO Integr. 2021, 2, 50.

[11] B. P. Dyett , H. Yu , J. Strachan , C. J. Drummond , C. E. Conn , Nat. Commun. 2019, 10, 4492. 31582802 10.1038/s41467-019-12508-8 PMC6776645

[12] H. Yu , J. S. Palazzolo , Y. Ju , B. Niego , S. Pan , C. E. Hagemeyer , F. Caruso , Adv. Healthcare Mater. 2022, 11, 2201151. 10.1002/adhm.202201151 36037807

[13] H. Kim , J. Sung , Y. Chang , A. Alfeche , C. Leal , ACS Nano 2018, 12, 9196. 30081623 10.1021/acsnano.8b03770 PMC6876307

[14] J. Zhai , C. Fong , N. Tran , C. J. Drummond , ACS Nano 2019, 13, 6178. 31082192 10.1021/acsnano.8b07961

[15] C. Fong , T. Le , C. J. Drummond , Chem. Soc. Rev. 2012, 41, 1297. 21975366 10.1039/c1cs15148g

[16] B. Patel , H. P. Thakkar , in Nanocarriers: Drug Delivery System, Springer Singapore, Singapore, 2021, pp. 227–254.

[17] T. G. Meikle , D. Dharmadana , S. V. Hoffmann , N. C. Jones , C. J. Drummond , C. E. Conn , J. Colloid Interface Sci. 2021, 587, 90. 33360913 10.1016/j.jcis.2020.11.124

[18] B. P. Dyett , H. Yu , S. Sarkar , J. B. Strachan , C. J. Drummond , C. E. Conn , ACS Appl. Mater. Interfaces 2021, 13, 53530. 34726885 10.1021/acsami.1c09909

[19] B. P. Dyett , H. Yu , B. Lakic , N. De Silva , A. Dahdah , L. Bao , E. W. Blanch , C. J. Drummond , C. E. Conn , J. Colloid Interface Sci. 2021, 600, 14. 34000474 10.1016/j.jcis.2021.03.161

[20] L. Boge , K. L. Browning , R. Nordström , M. Campana , L. S. E. Damgaard , J. Seth Caous , M. Hellsing , L. Ringstad , M. Andersson , ACS Appl. Mater. Interfaces 2019, 11, 21314. 31120236 10.1021/acsami.9b01826

[21] X. Hou , T. Zaks , R. Langer , Y. Dong , Nat .Rev. Mater. 2021, 6, 1078. 34394960 10.1038/s41578-021-00358-0 PMC8353930

[22] C. Zhang , Y. Ma , J. Zhang , J. C.‐T. Kuo , Z. Zhang , H. Xie , J. Zhu , T. Liu , Molecules 2022, 27, 1943. 35335310 10.3390/molecules27061943 PMC8949521

[23] R. Tenchov , R. Bird , A. E. Curtze , Q. Zhou , ACS Nano 2021, 15, 16982. 34181394 10.1021/acsnano.1c04996

[24] X. Han , H. Zhang , K. Butowska , K. L. Swingle , M.‐G. Alameh , D. Weissman , M. J. Mitchell , Nat. Commun. 2021, 12, 7233. 34903741 10.1038/s41467-021-27493-0 PMC8668901

[25] H. Yu , J. Iscaro , B. Dyett , Y. Zhang , S. Seibt , N. Martinez , J. White , C. J. Drummond , S. Bozinovski , J. Zhai , J. Am. Chem. Soc. 2023, 145, 24765. 10.1021/jacs.3c08729 37870621

[26] M. Gontsarik , A. Yaghmur , S. Salentinig , J. Colloid Interface Sci. 2021, 583, 672. 33039864 10.1016/j.jcis.2020.09.081

[27] L. Schoenmaker , D. Witzigmann , J. A. Kulkarni , R. Verbeke , G. Kersten , W. Jiskoot , D. J. A. Crommelin , Int. J. Pharm. 2021, 601, 120586. 33839230 10.1016/j.ijpharm.2021.120586 PMC8032477

[28] A. Gin , L. Dilay , J. A. Karlowsky , A. Walkty , E. Rubinstein , G. G. Zhanel , Expert Rev. Anti‐Infect. Ther. 2007, 5, 365. 17547502 10.1586/14787210.5.3.365

[29] M. Hurst , H. M. Lamb , L. J. Scott , D. P. Figgitt , Drugs 2002, 62, 2127. 12269858 10.2165/00003495-200262140-00013

[30] P. Savadi , T. Taghavi‐Fard , M. Milani , N. Hashemzadeh , V. Panahi , N. A. J. McMillan , S. Hallaj‐Nezhadi , Curr. Microbiol. 2020, 77, 2356. 32377819 10.1007/s00284-020-02008-0

[31] K. P. Fu , H. C. Neu , Antimicrob. Agents Chemother. 1978, 13, 358. 122519 10.1128/aac.13.3.358 PMC352246

[32] H. M. G. Barriga , M. N. Holme , M. M. Stevens , Angew. Chem. Int. Ed. 2019, 58, 2958. 10.1002/anie.201804067 PMC6606436 29926520

[33] P. Pushparaj Selvadoss , J. Nellore , M. Balaraman Ravindrran , U. Sekar , J. Tippabathani , Artif. Cells Nanomed. Biotechnol. 2018, 46, 268. 28362119 10.1080/21691401.2017.1307209

[34] B. Atef , R. A. H. Ishak , S. S. Badawy , R. Osman , J. Drug Delivery Sci. Technol. 2022, 67, 103032.

[35] M. Zewail , P. M. Passent , M. M. Ali , H. Abbas , Drug Delivery 2022, 29, 1663. 35616281 10.1080/10717544.2022.2079770 PMC9154769

[36] H. Yu , A. Angelova , B. Angelov , B. Dyett , L. Matthews , Y. Zhang , M. El Mohamad , X. Cai , S. Valimehr , C. J. Drummond , J. Zhai , Angew. Chem. 2023, 135, e202304977. 10.1002/anie.202304977 37391876

[37] H. Yu , B. Dyett , N. Kirby , X. Cai , M. El Mohamad , S. Bozinovski , C. J. Drummond , J. Zhai , Small 2024, 20, 2309200. 10.1002/smll.202309200 38295089

[38] J. Y. T. Chong , X. Mulet , L. J. Waddington , B. J. Boyd , C. J. Drummond , Langmuir 2012, 28, 9223. 22630595 10.1021/la301874v

[39] N. Mertz , A. Yaghmur , J. Østergaard , H. Amenitsch , S. W. Larsen , J. Colloid Interface Sci. 2021, 602, 415. 34144300 10.1016/j.jcis.2021.06.031

[40] A. Yaghmur , P. Laggner , M. Almgren , M. Rappolt , PLoS One 2008, 3, e3747. 19015726 10.1371/journal.pone.0003747 PMC2581612

[41] D. Demurtas , P. Guichard , I. Martiel , R. Mezzenga , C. Hébert , L. Sagalowicz , Nat. Commun. 2015, 6, 8915. 26573367 10.1038/ncomms9915 PMC4660369

[42] N. Tran , X. Mulet , A. M. Hawley , C. E. Conn , J. Zhai , L. J. Waddington , C. J. Drummond , Nano Lett. 2015, 15, 4229. 25984944 10.1021/acs.nanolett.5b01751

[43] L. Sagalowicz , S. Acquistapace , H. J. Watzke , M. Michel , Langmuir 2007, 23, 12003. 17949111 10.1021/la701410n

[44] N. Tran , X. Mulet , A. M. Hawley , T. M. Hinton , S. T. Mudie , B. W. Muir , E. C. Giakoumatos , L. J. Waddington , N. M. Kirby , C. J. Drummond , RSC Adv. 2015, 5, 26785.

[45] J. N. Israelachvili , D. J. Mitchell , B. W. Ninham , J. Chem. Soc., Faraday Trans. 2 1976, 72, 1525.

[46] J. Zhai , N. Tran , S. Sarkar , C. Fong , X. Mulet , C. J. Drummond , Langmuir 2017, 33, 2571. 28191966 10.1021/acs.langmuir.6b04045

[47] C. V. Kulkarni , Chem. Phys. Lipids 2019, 218, 16. 30476486 10.1016/j.chemphyslip.2018.11.004

[48] A. Scheeder , M. Brockhoff , E. N. Ward , G. S. Kaminski Schierle , I. Mela , C. F. Kaminski , J. Am. Chem. Soc. 2023, 145, 28240. 38085801 10.1021/jacs.3c11463 PMC10755748

[49] R. C. Hunter , T. J. Beveridge , Appl. Environ. Microbiol. 2005, 71, 2501. 15870340 10.1128/AEM.71.5.2501-2510.2005 PMC1087576

[50] W. H. Bowen , Odontology 2013, 101, 2.23224410

[51] L. A. Schneider , A. Korber , S. Grabbe , J. Dissemond , Arch. Dermatol. Res. 2007, 298, 413. 17091276 10.1007/s00403-006-0713-x

[52] T. K. Riddick , J. A. Bunger , W. B. Sakano , Organic Solvents:Physical Properties and Methods of Purification, John Wiley And Sons, Hoboken, NJ 1986.

[53] A. N. Schwier , N. Sareen , T. L. Lathem , A. Nenes , V. F. McNeill , J. Geophys. Res. 2011, 116, D16202.

[54] C. J. Drummond , F. Grieser , T. W. Healy , J. Chem. Soc., Faraday Trans. 1 1989, 185, 521.

[55] C. J. Drummond , F. Grieser , Langmuir 1987, 3, 855.

[56] J. Barauskas , M. Johnsson , F. Tiberg , Nano Lett. 2005, 5, 1615. 16089498 10.1021/nl050678i

[57] S. Salentinig , L. Sagalowicz , O. Glatter , Langmuir 2010, 26, 11670. 20578757 10.1021/la101012a

[58] C. Wang , X.‐L. Cao , L.‐L. Guo , Z.‐C. Xu , L. Zhang , Q.‐T. Gong , L. Zhang , S. Zhao , Colloids Surf., A 2016, 509, 601.

[59] E. F. Marques , R. O. Brito , Y. Wang , B. F. B. Silva , J. Colloid Interface Sci. 2006, 294, 240. 16125191 10.1016/j.jcis.2005.07.021

[60] E. Marques , A. Khan , M. Da Graca Miguel , B. Lindman , J. Phys. Chem. 1993, 97, 4729.

[61] A. B. Buya , B. A. Witika , A. M. Bapolisi , C. Mwila , G. K. Mukubwa , P. B. Memvanga , P. A. Makoni , C. I. Nkanga , Pharmaceutics 2021, 13, 2041. 34959323 10.3390/pharmaceutics13122041 PMC8708335

[62] B. Devrim , A. Bozkir , Nanostructures for Antimicrobial Therapy, Elsevier, Amsterdam, The Netherlands 2017, p. 169.

[63] D. Y. Wang , H. C. van der Mei , Y. Ren , H. J. Busscher , L. Shi , Front. Chem. 2020, 7, 1. 10.3389/fchem.2019.00872 PMC6965326 31998680

[64] M. Ferreira , M. Ogren , J. N. R. Dias , M. Silva , S. Gil , L. Tavares , F. Aires‐da‐Silva , M. M. Gaspar , S. I. Aguiar , Molecules 2021, 26, 2047. 33918529 10.3390/molecules26072047 PMC8038399

[65] Z. Drulis‐Kawa , A. Dorotkiewicz‐Jach , Int. J. Pharm. 2010, 387, 187. 19969054 10.1016/j.ijpharm.2009.11.033

[66] S. Bee , A. Banerjee , H. Önyüksel , J. Controlled Release 2012, 163, 34. 10.1016/j.jconrel.2012.06.002 22698939

[67] B. P. Dyett , S. Sarkar , H. Yu , J. Strachan , C. J. Drummond , C. E. Conn , ACS Appl. Mater. Interfaces 2024, 16, 24191. 38690584 10.1021/acsami.4c00921

[68] H. Yu , B. P. Dyett , J. Zhai , J. B. Strachan , C. J. Drummond , C. E. Conn , J. Colloid Interface Sci. 2023, 634, 279. 36542965 10.1016/j.jcis.2022.12.028

[69] X. Mulet , D. F. Kennedy , C. E. Conn , A. Hawley , C. J. Drummond , Int. J. Pharm. 2010, 395, 290. 20580796 10.1016/j.ijpharm.2010.05.029

[70] X. Mulet , B. J. Boyd , C. J. Drummond , J. Colloid Interface Sci. 2013, 393, 1. 23237762 10.1016/j.jcis.2012.10.014

[71] P. A. Lambert , J. R. Soc. Med. 2002, 95, 22. 12216271 PMC1308633

[72] J. Botelho , F. Grosso , L. Peixe , Drug Resist. Updates 2019, 44, 100640. 10.1016/j.drup.2019.07.002 31492517

[73] M. A. Bazán Henostroza , G. Diniz Tavares , M. Nishitani Yukuyama , A. De Souza , E. José Barbosa , V. Carlos Avino , E. dos Santos Neto , F. Rebello Lourenço , R. Löbenberg , N. Araci Bou‐Chacra , Int. J. Pharm. 2022, 621, 121782. 35489605 10.1016/j.ijpharm.2022.121782

[74] L. Boge , A. Umerska , N. Matougui , H. Bysell , L. Ringstad , M. Davoudi , J. Eriksson , K. Edwards , M. Andersson , Int. J. Pharm. 2017, 526, 400. 28476579 10.1016/j.ijpharm.2017.04.082

[75] F. Reck , A. Bermingham , J. Blais , A. Casarez , R. Colvin , C. R. Dean , M. Furegati , L. Gamboa , E. Growcott , C. Li , S. Lopez , L. Metzger , S. Nocito , F. Ossola , K. Phizackerley , D. Rasper , J. Shaul , X. Shen , R. L. Simmons , D. Tang , K. Tashiro , Q. Yue , ACS Infect. Dis. 2019, 5, 1045. 30861342 10.1021/acsinfecdis.9b00031

