# Supplementary material for: Ionizable Lipid Containing Nanocarriers for Antimicrobial Agent Delivery
Source: Small Sci. 2024 Aug 13;4(10):2400145. doi: 10.1002/smsc.202400145 (PMC11934984; doi:10.1002/smsc.202400145)
Supplement: Supplementary file 1 — Supplementary Material [file SMSC-4-2400145-s001.pdf]

Supporting Information

Ionizable lipid containing nanocarriers for antimicrobial agent delivery

Haitao Yu,[a,*] Sampa Sarkar,[a] Z. L. Shaw,[b] Xudong Cai,[a] Brendan Dyett,[a] Sue Lyn Yap,[a] Charlotte E. Conn,[a] Aaron Elbourne,[a] Calum J. Drummond,[a,*] Jiali Zhai [a,*]

a. School of Science, STEM College, RMIT University, Melbourne, Victoria 3000, Australia

b. School of Engineering, STEM College, RMIT University, Melbourne, Victoria 3000, Australia

Corresponding authors email:

Haitao Yu: haitao.yu@rmit.edu.au;

Calum J. Drummond: calum.drummond@rmit.edu.au;

Jiali Zhai: maggie.zhai@rmit.edu.au

LNP Characterization

Dynamic light scattering (DLS) and zeta potential measurements

DLS measurements of nanoparticles were performed at 25 °C on a Zetasizer APS (Malvern Panalytical, UK). The LNP solutions (with MO concentration of 50 mg/mL) were diluted by a factor of 10 with Milli-Q water before measurement. The refractive indices for MO and Milli-Q water were 1.46 and 1.33 respectively.[1,2] The Zetasizer software was used to determine the nanoparticle size ($Z_{average}$), intensity mean, and polydispersity index (PDI). For zeta potential measurements, samples were diluted to be 0.1 mg/mL MO concentration in 1 mM KNO_3 aqueous solution with desirable pH conditions, which were freshly adjusted by HNO_3/KOH immediately before the measurement and confirmed by a Mettler Toledo bench-top pH meter, or in H_2O condition using a Malvern Zetasizer Nano ZS (Malvern Instruments, UK) with polystyrene zeta potential cells (Malvern). All DLS and zeta potential measurements were repeated triplicate, n =3.

Small-angle X-ray scattering (SAXS)

SAXS experiments were proceeded using the SAXS/WAXS beamline at the Australian Synchrotron, ANSTO. A beam of wavelength λ = 1.033 Å (12.000 keV) with a dimension of 250 μm × 120 μm, a flux of 5×10^{12} photons s^{-1}, and an exposure time of 1 s were used. The distance from the sample to the detector (Pilatus 1-M) was 1.6 m, with a corresponding q range of 0.01−0.5 $Å^{-1}$. The scattering vector, q, is defined by $q = 4\pi\sin\theta/\lambda$ where θ is the scattering angle and λ is the wavelength. Sample solutions with MO concentration of 50 mg/mL were transferred into 96-well plates and sealed before being mounted in the beam path. The data were analyzed using Scatterbrain Software.[2] 1D SAXS scattering patterns of samples were plotted by extracting the intensity of the corresponding 2D SAXS scattering images. According to Bragg's law, the spacing d was calculated as $d = 2\pi/q$. And the lattice parameter (a) of the mesophases was obtained based on the following equations:$a = d(h^2 + k^2 + l^2)^{1/2}$ for cubic phases; $a = (2/\sqrt{3}) * d(h^2 + k^2 + hk)^{1/2}$ for hexagonal phase, where (h,k,l) are Miller indices assigned to the mesophase.

Cryogenic transmission electron microscopy (cryo-TEM)

LNPs were visualized in their native frozen hydrated state using cryogenic transmission electron microscopy. The samples were vitrified on a lacey carbon film coated copper grids (ProSciTech) and vitrified using an FEI Vitrobot. The samples were vitrified with a humidity of 95 %, blot force of 4, and blot time of 4 s. The grids were then transferred with a Gatan 626 cryo-transfer specimen holder into the FEI Tecnai F30 Transmission Electron Microscope, and imaged at a defocus level of −5 μm. Fast Fourier transform (FFT) analysis was used for identification of crystallographic structures present in the LNPs by FFT analysis in ImageJ software.

TNS assay

The degree of ionization of LNPs was determined using a TNS assay to evaluate the pK_a^{app} of ALC-0315.[3–5] LNPs were mixed with various buffer solutions and TNS-dissolved DMSO solution to make the final solutions with a lipid concentration of 0.5 mg/mL and 2 μM TNS in 10 mM buffers with pH of 3–11 (citrate buffer for pH 3 – 6, sodium phosphate buffer for pH 6 – 8, and sodium carbonate buffer for pH 9 – 11). The final solution was incubated for 15 min at room temperature (shielded from light) and then the fluorescence intensity (FI, excitation: 325 nm and emission: 435 nm) was measured using a PerkinElmer Multimode EnSight plate reader. The normalization of FI was obtained by employing the equation, $Normalized\ FI =$

$(FI - FI_{pH11})/(FI_{pH3} - FI_{pH11})$. pK_a^{app} of ALC-0315, representing the pH at which 50% of the ionizable lipid population in LNPs are protonated, was obtained by fitting the normalized FI using Absolute IC50 Model in GraphPad Prism Software.

References

[1] H. Yu, J. S. Palazzolo, Y. Ju, B. Niego, S. Pan, C. E. Hagemeyer, F. Caruso, *Adv Healthc Mater* **2022**, *11*, 2201151.

[2] H. Yu, B. P. Dyett, J. Zhai, J. B. Strachan, C. J. Drummond, C. E. Conn, *J Colloid Interface Sci* **2023**, *634*, 279.

[3] S. A. Dilliard, Q. Cheng, D. J. Siegwart, *Proceedings of the National Academy of Sciences* **2021**, *118*, 52.

[4] M. Kim, M. Jeong, S. Hur, Y. Cho, J. Park, H. Jung, Y. Seo, H. A. Woo, K. T. Nam, K. Lee, H. Lee, *Sci Adv* **2021**, *7*, eabf4398.

[5] P. Patel, N. M. Ibrahim, K. Cheng, *Trends Pharmacol Sci* **2021**, *42*, 448.

Supplementary Figures and Tables

Table S1. $Z_{average}$ size, polydispersity index (PDI), and intensity mean of MO-ALC LNPs using DLS measurement

Samples	$Z_{average}$ (nm)	PDI	Intensity mean (nm)
MO	230 ± 2	0.15 ± 0.01	268 ± 98
MO-1%ALC	222 ± 2	0.20 ± 0.01	277 ± 118
MO-2%ALC	245 ± 5	0.26 ± 0.03	339 ± 194
MO-5%ALC	277 ± 5	0.27 ± 0.02	373 ± 201
MO-10%ALC	317 ± 7	0.31 ± 0.04	363 ± 101

Table S2. $Z_{average}$ size, polydispersity index (PDI), intensity mean, zeta potential (ZP), and pH of LNPs and Pip@LNPs in water[#]

Samples	$Z_{average}$ (nm)	PDI	Intensity mean (nm)	ZP (mV)	pH
MO	230 ± 2	0.15 ± 0.01	268 ± 98	-21 ± 5	5.1
MO-5%ALC	277 ± 5	0.27 ± 0.02	373 ± 201	34 ± 5	6.1
MO-5%ALC-1%OA	269 ± 6	0.28 ± 0.02	367 ± 198	20 ± 4	6.2
MO-5%ALC-2%OA	270 ± 3	0.28 ± 0.02	355 ± 156	14 ± 4	6.1
MO-5%ALC-5%OA	246 ± 4	0.24 ± 0.02	326 ± 138	3 ± 6	5.7
MO-1%OA	266 ± 1	0.15 ± 0.05	294 ± 104	-23 ± 11	5.0
MO-2%OA	276 ± 5	0.16 ± 0.03	331 ± 124	-25 ± 5	4.9
MO-5%OA	278 ± 1	0.17 ± 0.01	334 ± 126	-26 ± 5	4.8
Pip@MO	251 ± 5	0.19 ± 0.03	312 ± 140	-25 ± 4	3.9
Pip@MO-5%ALC	240 ± 6	0.26 ± 0.02	331 ± 160	21 ± 6	5.2

Pip@MO-5%ALC-1%OA	287 ± 4	0.24 ± 0.01	380 ± 167	9 ± 4	5.2
Pip@MO-5%ALC-2%OA	292 ± 5	0.24 ± 0.02	377 ± 173	11 ± 4	5.1
Pip@MO-5%ALC-5%OA	287 ± 9	0.22 ± 0.01	367 ± 149	-4 ± 4	4.5
Pip@MO-1%OA	261 ± 1	0.16 ± 0.04	303 ± 128	-28 ± 5	3.6
Pip@MO-2%OA	265 ± 5	0.13 ± 0.04	293 ± 96	-32 ± 6	3.6
Pip@MO-5%OA	266 ± 1	0.17 ± 0.01	307 ± 120	-34 ± 5	3.6

#As a comparison, the pH values of mill-q water and F127 aqueous solution (5 mg/mL) in air are 5.7 and 5.6, respectively.

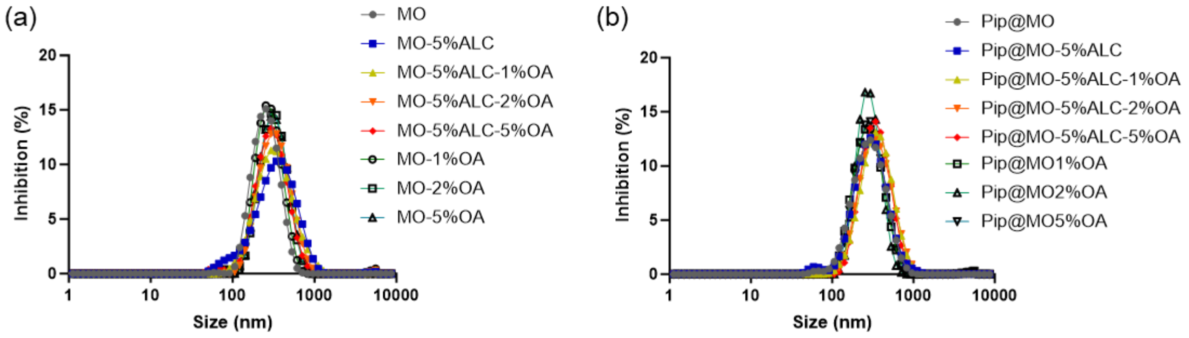

Figure S1. Intensity profile of LNPs and Pip@LNPs in water using DLS measurement.

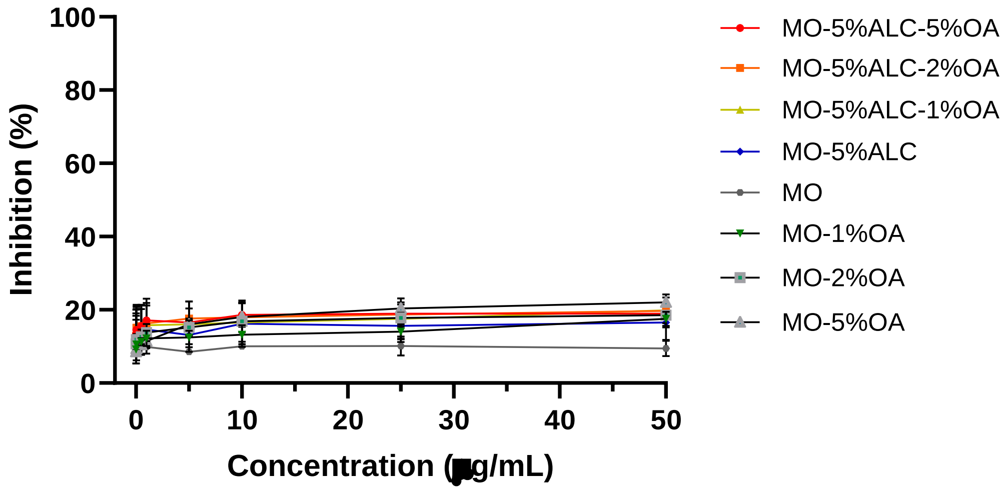

Figure S2. Antimicrobial activity (inhibition, %) of empty LNPs (as negative control) for Gram-negative *P. aeruginosa* for 24-hour incubation at 37°C, n = 3.

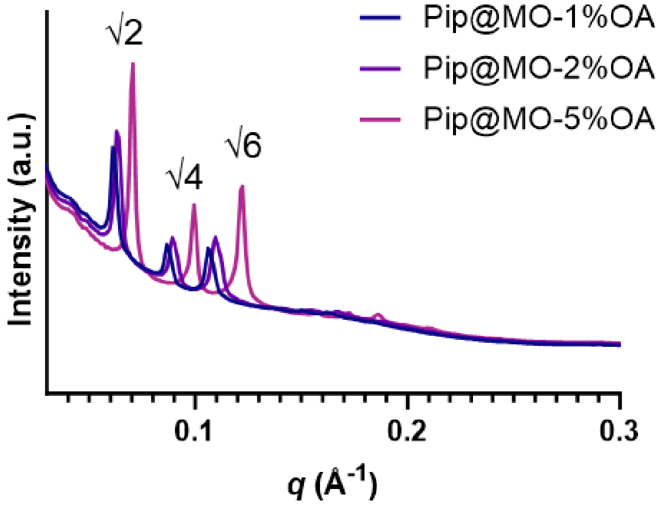

Figure S3. SAXS diffraction patterns of Pip@MO-OA samples with the molar concentrations (1, 2, and 5%) of OA in water at room temperature 25 °C.
